# MLP-PSO Hybrid Algorithm for Heart Disease Prediction

**DOI:** 10.3390/jpm12081208

**Published:** 2022-07-25

**Authors:** Ali Al Bataineh, Sarah Manacek

**Affiliations:** 1 Department of Electrical and Computer Engineering, Norwich University, Northfield, VT 05663, USA; 2 Department of Nursing, College of Nursing and Health Sciences, The University of Vermont, Burlington, VT 05405, USA; smanacek@norwich.edu

**Keywords:** heart disease prediction, machine learning, neural networks, MLP, PSO

## Abstract

Background: Machine Learning (ML) is becoming increasingly popular in healthcare, particularly for improving the timing and accuracy of diagnosis. ML can provide disease prediction by analyzing vast amounts of healthcare data, thereby, empowering patients and healthcare providers with information to make informed decisions about disease prevention. Due to the rising cost of treatment, one of the most important topics in clinical data analysis is the prediction and prevention of cardiovascular disease. It is difficult to manually calculate the chances of developing heart disease due to a myriad of contributing factors. Objective: The aim of this paper is to develop and compare various intelligent systems built with ML algorithms for predicting whether a person is likely to develop heart disease using the publicly available Cleveland Heart Disease dataset. This paper describes an alternative multilayer perceptron (MLP) training technique that utilizes a particle swarm optimization (PSO) algorithm for heart disease detection. Methods: The proposed MLP-PSO hybrid algorithm and ten different ML algorithms are used in this study to predict heart disease. Various classification metrics are used to evaluate the performance of the algorithms. Results: The proposed MLP-PSO outperforms all other algorithms, obtaining an accuracy of 84.61%. Conclusions: According to our findings, the current MLP-PSO classifier enables practitioners to diagnose heart disease earlier, more accurately, and more effectively.

## 1. Introduction

Machine Learning, or ML, is becoming increasingly popular in the medical field, particularly in diagnostics and treatment management [1,2]. There has been a great deal of research into how ML can improve both the timing and accuracy of diagnosis [3]. Accurate diagnosis is a critical component of global healthcare systems. In the United States, approximately 5% of outpatients receive an incorrect diagnosis for serious medical conditions [4]. This not only poses a risk of patient harm but also creates inefficiencies in the healthcare system, such as inappropriate diagnostic testing [5]. Diagnostic errors increase healthcare spending and create mistrust of the healthcare system.

Additionally, there is growing dissatisfaction among healthcare providers about the amount of time spent inputting and analyzing data on the computer, which detracts from the time spent face-to-face with patients [6]. In recent years, Artificial-Intelligence (AI) tools built with ML algorithms have emerged as powerful diagnostic aids. ML algorithms are useful for predicting the outcome of existing data, and, in the healthcare industry, there is a massive amount of data.

Data mining converts a large amount of raw healthcare data into information that can be used to make more informed decisions and predictions [7]. ML can use the information to provide assistance with decision-making and prediction [8]. These predictions provide critical insight in advance, which ultimately enables patients and healthcare providers to take action to prevent the development of illness altogether.

By quickly providing more precise predictions and diagnoses, ML technology has the potential to revolutionize healthcare. ML methods, with advances in telehealth technology, could increase access to high-quality healthcare for underserved populations worldwide. It holds the potential for increasing the time spent face-to-face with patients. ML technology may also reduce unnecessary diagnostic testing and overall healthcare spending by improving the accuracy of diagnosis.

When compared to established processes, ML tools have significantly improved a number of measurement values and have increased the speed and objectivity of the analyses [9]. The rates of one complex disease, in particular, could be greatly reduced from early prevention interventions. Heart disease, also known as cardiovascular disease, is a leading cause of morbidity and mortality worldwide and is considered a global epidemic [10,11]. Cardiovascular disease is an umbrella term that includes any disease of the heart or blood vessels.

Examples of cardiovascular diseases include atherosclerosis (plaque build up in an artery), coronary artery disease, heart rhythm abnormalities (arrhythmias), congenital heart defects, and heart valve abnormalities [12]. These conditions may lead to further complications, such as stroke, heart attack, chest pain (angina), and heart failure [13]. According to the Centers for Disease Control and Prevention (CDC), heart disease is the leading cause of death for both men and women in the United States. The following is stated by the CDC and the New York State Department of Health:

- Someone in the United States dies from cardiovascular disease every 34 seconds.
- Every year, approximately 610,000 people in the United States die from heart disease, accounting for one out of every four deaths.
- For both men and women, heart disease is the leading cause of death. In 2009, men accounted for more than half of all heart disease deaths.
- Coronary Heart Disease (CHD) is the most common type of heart disease, claiming the lives of over 370,000 people each year.
- Every year, approximately 735,000 Americans suffer a heart attack. A total of 525,000 of these are first-time heart attacks, while 210,000 occur in people who have already had a heart attack.

The cost of treatment for heart failure alone in 2020 was about $43.6 billion US dollars and is projected to reach $69.7 billion US dollars by 2030 [14]. Thus, one of the most important topics in clinical data analysis is the prediction and prevention of cardiovascular disease. However, it is difficult to manually calculate the chances of developing heart disease due to a number of contributing risk factors, such as diabetes, high cholesterol, abnormal pulse rate, high blood pressure, and a variety of other factors [15]. Fortunately, ML can be used to predict the presence or absence of numerous diseases, such as Alzheimer’s Disease, cancer, stroke, heart disease, diabetes, and liver disease by analyzing massive amounts of medical data [16,17,18].

Researchers have proposed a number of ML techniques to analyze massive amounts of complex medical data, assisting healthcare professionals in predicting heart disease [19,20]. In [21], the authors developed a Bayesian network model for predicting heart disease. They employed two different Bayesian classifier implementations—namely the Bayesian Belief Network and the Naive Bayes. They concluded that the Bayesian Belief network provided better performance than the Naïve Bayes in predicting heart diseases. The authors in [22], utilized Decision Tree, Naïve Bayes, K-Nearest Neighbor (KNN), and bagging algorithms. KNN was discovered to be the most effective at detecting heart disease.

The authors in [23] developed an AI-based heart detection system with higher accuracy using the random forest classifier. In [24], the authors proposed various machine-learning algorithms and deep learning for detecting heart disease. Good results with a deep-learning approach were obtained and validated using metrics, such as the accuracy and a confusion matrix. The authors in [25] developed a variety of machine-learning algorithms to identify and diagnose cardiac disease.

Several feature selection algorithms were used to remove irrelevant and noisy data from the extracted feature space, and the results of each feature selection algorithm, as well as classifiers, were evaluated using metrics, such as the AUC and F1-score. The authors in [26] presented a multilayer perceptron (MLP) neural network and support vector machine (SVM) models for developing a decision support system for heart disease diagnosis.

The results showed that the MLP outperformed the SVM. In [27], the authors introduced an expert system that stacked two SVM models for the effective prediction of heart failure in order to assist cardiologists in improving the diagnosis process. In [28], the authors presented an efficient hybrid neuroevolution model that combined MLP and a multi-verse optimizer for heart disease detection and achieved a good detection rate. The authors in [29] presented a hybrid decision support system for heart disease classification based on SVM and integer-coded genetic algorithm (GA).

Integer-coded GA is employed for selecting the important and relevant features and discarding the irrelevant and redundant ones to maximize SVM’s classification accuracy. The authors of [30] presented a hybrid approach to detecting heart disease based on feature selection, fuzzy-weighted pre-processing, and an artificial immune recognition system. According to the findings, the proposed method can be used in medical decision support systems.

This paper proposes a swarm-based MLP (MLP-PSO) network for heart disease prediction. The particle swarm optimization (PSO) algorithm is used as an efficient optimization technique to guide MLP training optimization, which includes determining the optimal weights and bias values. It has been demonstrated that the PSO algorithm provides better performance when compared to other heuristic optimization methods [31,32,33]. PSO is simple to implement, computationally efficient, and robust in terms of the controlling parameters [34,35].

The performance of the MLP-PSO is compared against several cutting-edge ML algorithms using well-known performance evaluation metrics. The algorithms included are: (1) GaussianNB Classifier, (2) Logistic Regression Classifier, (3) Decision Tree Classifier, (4) Random Forest Classifier, (5) Gradient Boosting Classifier, (6) K-Nearest Neighbors Classifier, (7) XGB Classifier, (8) Extra Trees Classifier, (9) Support Vector Classifier, and (10) Multilayer Perceptron Classifier with BP. The Cleveland Heart Disease dataset from the UCI Repository is benchmarked to evaluate the performance of all algorithms.

The dataset comprises 13 features and one target variable (0 or 1). The task is to apply the ML algorithms including the proposed MLP-PSO to predict whether a person will develop a heart disease (1) or not (0) based on the 13 medical features provided in the Cleveland Heart Disease dataset. Our proposed methodologies can be used to to predict the development of cardiovascular disease so that patients and healthcare providers will have the information necessary to make patient-centered decisions, take primary prevention measures sooner, and ultimately improve patients’ quality of life.

The remainder of this paper is structured as follows. Section 2 discusses the materials and methods employed in this research. Experiments and results are described in Section 3. Finally, our conclusions and future work directions of this research are given in Section 4.

## 2. Materials and Methods

### 2.1. Dataset

The dataset used in this research is the Cleveland Heart Disease dataset [36]. It is an imbalanced classification dataset consisting of 303 instances. The dataset contains 13 features and one target variable, which are described below.

1. **Age:** indicates the age of the individual.
2. **Sex:** displays the individual’s gender in the following format:
  - 0 = female.
  - 1 = male.
3. **Chest-pain type:** displays the individual’s type of chest-pain in the following format:
  - 1 = typical angina.
  - 2 = atypical angina.
  - 3 = non-anginal pain.
  - 4 = asymptotic.
4. **Resting Blood Pressure:** displays an individual’s resting blood pressure in mmHg (unit).
5. **Serum Cholesterol:** displays the serum cholesterol in mg/dL (unit).
6. **Fasting Blood Sugar:** compares the fasting blood sugar value of an individual with 120 mg/dL. If fasting blood sugar >120 mg/dL then: 1 (true) else: 0 (false).
7. **Resting ECG:** displays resting electrocardiographic results:
  - 0 = normal.
  - 1 = having ST-T wave abnormality.
  - 2 = left ventricular hypertrophy.
8. **Max heart rate achieved:** displays an individual’s maximum heart rate attained.
9. **Exercise-induced angina:**
  - 0 = no.
  - 1 = yes.
10. **ST depression induced by exercise relative to rest:** displays the value, which can be an integer or float.
11. **Peak exercise ST segment:**
  - 1 = upsloping.
  - 2 = flat.
  - 3 = downsloping.
12. **Number of major vessels (0–3) colored by fluoroscopy:** displays the value as integer or float.
13. **Thal:** displays the thalassemia:
  - 3 = normal.
  - 6 = fixed defect.
  - 7 = reversible defect.
14. **Diagnosis of heart disease:** Displays whether the individual is suffering from heart disease or not:
  - 0 = absence.
  - 1 = present.

#### 2.1.1. Exploratory Data Analysis

Exploratory Data Analysis, or EDA, is a critical process of conducting preliminary investigations on data in order to identify patterns, relationships between variables, and outliers; test hypotheses; and validate assumptions using summary statistics and graphical representations. EDA is a significant step in fine-tuning the given dataset in a different form of analysis to understand the insights of the key characteristics of the dataset’s various entities. It paints a clear picture of the features and their relationships. Figure 1 depicts a visual representation of the correlation matrix. A correlation matrix is a table that displays the correlation coefficient between two or more variables.

To calculate the correlation coefficient/Pearson correlation coefficient, each attribute of the dataset is compared to the other attributes. This analysis shows which pairs have the highest correlation. As highly correlated pairs represent the same variance of the dataset, we can further analyze them to determine which attribute among the pairs is most important for building the model. Correlation values range from −1 to +1, with −1 indicating the total negative linear correlation, with 0 indicating no correlation and +1 indicating total positive correlation.

It is clear that no single feature in the dataset has a high correlation with the target value. Furthermore, some of the features are negatively correlated with the target value, while others are positively correlated. In mathematical terms, the Pearson correlation coefficient between two variables, *x* and *y*, can be calculated using the following formula:

(1) $$r=\frac{\sum \left(x_{i}-\bar{x}\right)\left(y_{i}-\bar{y}\right)}{\sqrt{\sum {\left(x_{i}-\bar{x}\right)}^{2}\sum {\left(y_{i}-\bar{y}\right)}^{2}}}$$

where *r* is the Pearson correlation coefficient, $x_{i}$ represents different values of the *x*-variable in a sample, $\bar{x}$ is the mean of the values of the *x*-variable, $y_{i}$ represents different values of the *y*-variable in a sample, and $\bar{y}$ is the mean of the values of the *y*-variable.

The histogram for each attribute in the dataset is depicted in Figure 2. A histogram shows the distribution of a specific attribute. Looking at the histograms, we can see that each attribute has a different distribution range. As a result, prior to feeding data into machine-learning models, normalizing the input attribute (features) should be extremely beneficial.

A bar plot for the target class is shown in Figure 3. It is critical that the dataset on which we are working is approximately balanced. That is, the target classes are roughly equal in size. A dataset that is extremely unbalanced can render the entire model training. As illustrated in Figure 3, the ratio between the two classes is not exactly 50%; however, it is sufficient to proceed without reducing or expanding our dataset.

#### 2.1.2. Data Preprocessing

Data preprocessing is a critical step in ML that involves cleaning and organizing the raw data in order to make it suited for building and training ML models. Real-world data is frequently incomplete, inconsistent, and/or lacking in specific behaviors or trends, as well as containing numerous errors. Data preprocessing, in simple terms, is a data-mining technique that transforms raw data into a readable and understandable format. After conducting preliminary data investigations with EDA, we followed established steps to ensure that our data was successfully preprocessed.

1. **Identifying and handling missing/null values**
  It is critical to correctly identify and handle missing values in data preprocessing; otherwise, we may draw incorrect and erroneous conclusions and inferences from our data. It was observed that there are 6 rows out of the 303 rows containing null values, with four belonging to the variable ‘**ca**’ and two belonging to the variable ‘**thal**’. There are two ways to deal with null values: drop or impute. The former is not completely efficient, and it is recommended that we use it only when the dataset has sufficient samples, and we must ensure that there is no additional bias after deleting the data. As a result, we applied the latter and imputed the mean in place of the null values because the null values are few. In this case, we compute the mean of a specific feature, such as ca, that contains a missing value and replace the result for the missing value with the mean. This method can add variance to the dataset and efficiently negate any data loss. Hence, it provides more accurate results than the first method (drop).
2. **Categorical data encoding**
  Categorical data is information that is divided into distinct categories within a dataset. There are some categorical variables in the heart disease dataset: ‘**sex**’, ‘**cp**’, ‘**fbs**’, ‘**restecg**’, ‘**exang**’, ‘**slope**’, ‘**ca**’ and ‘**thal**’. Mathematical equations serve as the foundation for ML models. As a result, we can intuitively understand that keeping categorical data in the equation will cause problems because the equations only involve numeric values. For this reason, we converted them to numerical values.
3. **Splitting the dataset into training and testing sets**
  This step in data preprocessing involves taking the dataset and dividing it into two separate sets. The training dataset is the first subset, which is used to fit or train the model. The second subset, which is referred to as the test dataset, is used is to validate the fit model. In general, we divide the data set into a 70:30 or 80:20 ratio, which means that 70% or 80% of the data is used to train or fit the ML model, and 30% or 20% is used to test or evaluate the trained ML model [37]. However, depending on the shape and size of the data set, the splitting ratio can be changed.
4. **Feature scaling**
  In data preprocessing, feature scaling is a method for standardizing the independent variables of a dataset within a specific range. To put it another way, feature scaling narrows the range of the independent variables so that we can compare them on common grounds. In the heart disease dataset, we have the variables ‘**age**’, ‘**restbp**’, ‘**chol**’, ‘**thalach**’, ‘**oldpeak**’ that do not have the same scale. In such a case, if we compute any two values from the ‘**restbp**’ and ‘**chol**’ columns, the ‘**chol**’ values will dominate the ‘**restbp**’ values and produce incorrect results. Therefore, we must perform feature scaling in order to eliminate this issue. This is critical for ML algorithms, such as logistic regression, MLP neural networks, and others that use gradient descent as an optimization technique that necessitates data scaling. Furthermore, distance algorithms, such as SVM and KNN, are most influenced by the range of the features. This is due to the fact that they use distances between data points to determine their similarity behind the scenes. The two most-discussed methods to perform feature scaling are Normalization and Standardization.
  - **Normalization**
    Normalization is a scaling technique that shifts and rescales values so that they end up ranging between 0 and 1. Min–Max scaling is another name for it. The Normalization equation is written mathematically as
    (2) $$X^{\prime }=\frac{X-X_{min}}{X_{max}-X_{min}}$$
    Here, $X_{min}$ and $X_{max}$ are the minimum and the maximum values of the feature, respectively.
  - **Standardization**
    Standardization is a scaling technique in which values are centered around the mean with a unit standard deviation. This means that the features will be rescaled to ensure the mean and the standard deviation are 0 and 1, respectively. For our work, we used the standardization method. The standardization equation is as follows:
    (3) $$X^{\prime }=\frac{X-\;\mu }{\sigma }$$
    Here, μ is the mean of the feature values, and σ is the standard deviation of the feature values.

### 2.2. Methodology of the Proposed System

#### 2.2.1. MLP

MLP is a complex function that accepts numerical inputs and produces numerical outputs [38]. Figure 4 depicts a fully connected MLP network. It is made up of three layers: the input layer takes raw input from the domain, the hidden layer extracts features, and the output layer makes a prediction. A deep neural network is has more than one hidden layer [39]. Adding more hidden layers, on the other hand, can result in vanishing gradient problems, which necessitates the use of special algorithms to resolve. The number of hidden layers and the number of hidden neurons are referred to as hyperparameters of the MLP, and they must be carefully chosen.

To find ideal values for these hyperparameters, cross-validation techniques [40] are frequently used. Hidden and output neurons in the MLP networks employ activation functions (*f*). Typically, all hidden neurons use the same activation function. The output layer, on the other hand, will typically use a different activation function than the hidden layers. The choice is determined by the model’s goal or type of prediction. The purpose of an activation function is to add non-linearity to the neural network [41].

Sigmoid and ReLU are two of the most popular activation functions used in neural networks [42]. As shown in Figure 5, the sigmoid function, also known as the logistic function, takes any real value as input and outputs values in the range of 0 to 1. The function provides a smooth gradient and is differentiable. The rectified linear activation function, or ReLU, outputs zero if it receives any negative input, otherwise, it will output the same value. The ReLU function is often used in hidden layers because it is computationally efficient [43].

The outputs of the MLP network are determined by weights and bias values as well as the inputs. In most cases, we want to create an MLP that can make predictions, such as predicting heart disease (0 = no and 1 = yes) based on medical data, such as a person’s age, serum cholesterol, fasting blood sugar, and so on. To accomplish this, we use data with known input and output values to determine a set of weights and bias values so that the MLP generates computed outputs that closely match the known outputs. This process is called training the MLP network.

Backpropagation (BP) [44] is frequently used for training. After training with data with known outputs, the network can be presented with new data, and predictions can be made. However, there is a hard requirement for BP to work properly. The cost function and the activation function, for instance, sigmoid, must be differentiable. Furthermore, BP might converge to sub-optimal weights and biases from which it cannot escape. PSO algorithms are a powerful technique that can guide training optimization.

#### 2.2.2. PSO

Nature has always been a source of inspiration for researchers to develop new computing methodologies by observing how naturally occurring phenomena behave in various environmental situations to solve complex real-world problems [45,46]. This has led to groundbreaking analysis that has given rise to new fields, such as artificial immune systems [47], evolutionary computation [48], and swarm intelligence [49]. PSO is an adaptive strategy and a global optimization technique [50] that has been successfully applied for solving search and optimization problems in various domains.

However, unlike genetic algorithms, PSO is simple and easy to implement and has fewer parameters to adjust. PSO belongs to the swarm intelligence field. Swarm intelligence is the study of computational systems inspired by the collective intelligence that arises from the cooperation of homogeneous agents (e.g., bird flocks and fish schools) in the environment. PSO is inspired by the organized behavior of bird flocking and fish schooling [51].

The PSO algorithm runs by maintaining a population of candidate solutions in the search space. In each iteration, every candidate solution is evaluated by the objective function being optimized, measuring the fitness of that solution. All candidate solutions can be thought of as particles “flying” through the fitness landscape, seeking to find the objective function’s maximum or minimum [52]. Algorithm 1 provides a pseudocode listing of the PSO algorithm.   
**Algorithm 1:** Pseudocode for the PSO algorithm [53].   **Input**: $Population_{size}$   **Output**: $P_{g\_best}$   Population ← ∅;   $P_{g\_best}$ ← ∅;   **for** i=1 **to** $Population_{size}$ **do**
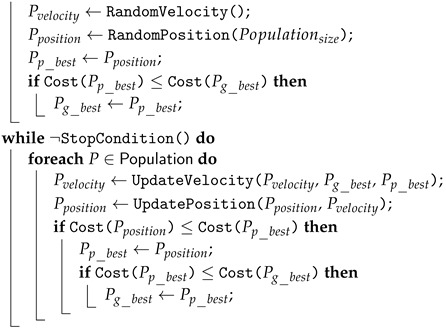
   **return** $P_{g\_best}$

Initially, the PSO algorithm randomly selects a population of candidate solutions within the search space. Figure 6 shows the initial state of two particles searching for the global minimum in a one-dimensional search space. The search space consists of all the possible solutions along the *x*-axis; the curve shows the objective function. The PSO algorithm has no knowledge of the primary objective function and hence has no means of identifying if any of the candidate solutions are near to or far from the local or global minimum.

The algorithm essentially applies the objective function to evaluate its candidate solutions and acts on the basis of the resulting fitness values. Each individual particle maintains its position, consisting of the candidate solution, its measured fitness, and its velocity. In addition, it remembers the best fitness value obtained thus far during the procedure of the algorithm, described as the individual best fitness, and the candidate solution that achieved this fitness is described as the individual best position or individual best candidate solution. In the end, the PSO algorithm maintains the best fitness value obtained among all particles, called the global best fitness, and the candidate solution that achieved this fitness is called the global best position or global best candidate solution.

The PSO algorithm iteratively performs three simple steps until some stopping criterion is reached. These are [54]:

1. Evaluate the fitness of each particle or candidate solution in the population using the objective function being optimized.
2. Update the best individual and global fitnesses and positions by comparing the newly evaluated fitnesses with the prior best individual and global fitnesses and replacing the best fitnesses and positions as needed.
3. Update the velocity and position of every particle in the population. This updating step is responsible for the optimization ability of the PSO algorithm.

#### 2.2.3. Training MLP Using PSO

This subsection discusses using the biologically inspired PSO algorithm to train MLP neural networks for heart disease prediction. A schematic diagram of the proposed methodology is shown in Figure 7. The PSO algorithm is a metaheuristic that can search through extremely large spaces of candidate solutions. When training MLP networks, the weights and biases values are adjusted using an error function as our optimization objective. The goal is to find the values of the weights and biases that minimize the error function. Each particle in the swarm population has a virtual position associated with it that represents a possible solution to a minimization problem.

In the case of MLP networks, the position of a particle represents the network’s weights and biases. The objective is to find a combination of positions/weights that causes the network to produce computed outputs that match the outputs of the labeled training data. To train an MLP network, the PSO algorithm uses a population of potential solutions called a swarm. Each potential solution in the swarm is referred to as a particle. As we want to optimize the MLP using PSO, each particle’s position is a vector that represents the values for the MLP’s weights and biases. For example, a 14-input, 30-hidden, one-output MLP network is instantiated for the Cleveland heart disease dataset.

A fully-connected 14–30–1 MLP network will have (14*30)+(30*1)+(30+1)=811 weights and bias values or 811 dimensions for each particle position in the swarm (Appendix A contains an example of how to represent the MLP training problem using PSO). These weights and bias values are randomly generated for each particle at the beginning of the training. Furthermore, each particle has three properties that determine its direction: the current velocity, the local best solution, and the global best solution.

The PSO algorithm also includes three major constants: and cognitive coefficient ($c_{1}$), social coefficient ($c_{2}$), and inertia weight (*w*). Each of these constants is related to the local best solution, the global best solution, and the velocity of the particles. The computation of a particle’s new velocity and position is the key to the PSO algorithm. The velocity and position update equations are as follows.

(4) $$v_{t+1}=w*\;v_{t}+c_{1}*\;r_{1}*\left(pbest_{t}-x_{t}\right)+c_{2}*\;r_{2}*\left(gbest_{t}-x_{t}\right)$$

where $v_{t+1}$ is the new velocity at time t+1, $v_{t}$ is the current velocity at time *t*, $r_{1}$ and $r_{2}$ are random variables in the range [0,1), $pbest_{t}$ is the particle’s best position found thus far, $x_{t}$ is the particle’s current position, and $gbest_{t}$ is the global best position in the swarm thus far. Once the new velocity, $v_{t+1}$, has been determined, it is used to compute the new particle position $x_{t+1}$ as follows.

(5) $$x_{t+1}=x_{t}+v_{t+1}$$

### 2.3. Supervised Machine Learning Algorithms

In this study, ten supervised ML algorithms are developed for comparison with our proposed MLP-PSO.

#### 2.3.1. Logistic Regression

Logistic Regression is a technique for estimating discrete binary values (such as 0/1 and true/false) from a given set of independent variables. It predicts the likelihood of an event by fitting data to a logit function. It always outputs numbers between 0 and 1 [55].

#### 2.3.2. Support Vector Machine

The Support Vector Machine (SVM) algorithms aim to find a hyperplane with the maximum margin, i.e., the best that separates data points by their class, either class 1 or class 0 in an n-dimensional space (where n is the number of used features of the dataset) [56].

#### 2.3.3. KNN (K-Nearest Neighbors)

KNN is a simple type of algorithm that requires no learning because it has no model other than storing the dataset. KNN uses the training dataset to classify new instances. A new instance is classified by determining which of the K instances (neighbors) in the training dataset are the most similar to it and assigning it a class by a majority vote [57]. To find the K instances that are the most similar to a new instance, a distance function, such as Euclidean [58] is frequently used.

#### 2.3.4. Decision Tree

Decision Tree is an easy-to-implement algorithm that works well for both continuous and categorical dependent variables. The algorithm splits the population into two or more homogeneous sets based on the most significant features in order to create groups that are as distinct as possible [59].

#### 2.3.5. Random Forest

Random Forest is an ensemble learning method that constructs several decision trees referred to as a “forest”. Each tree is classified, and the tree “votes” for that class in order to classify a new object based on its features. The classification with the most votes is chosen by the forest. During tree construction, it employs the random subspace method and bagging. It has feature importance built in [60].

#### 2.3.6. Extra Trees Classifier

Extra Trees (Extremely Randomized Trees) is an ensemble learning algorithm as well. It builds a set of decision trees. The decision rule is chosen at random during tree construction [61]. Except for the random selection of split values, this algorithm is similar to Random Forest.

#### 2.3.7. Gradient Boosting

Gradient Boosting is an ensemble learning algorithm that constructs a strong model from a collection (ensemble) of weaker models. The algorithm constructs a strong model by learning from each of the weak learners iteratively [62].

#### 2.3.8. Naive Bayes

Naive Bayes (NB) is a probabilistic model based on Bayes’ Theorem that makes a strong (naive) assumption of independence among features. A Gaussian NB algorithm is a special type of NB algorithm, specifically used for continuous variables (features). The algorithm assumes that all the features are following a Gaussian distribution [63].

#### 2.3.9. XGB

XGB (eXtreme Gradient Boosting) is a gradient-boosted decision tree ensemble algorithm designed to produce superior results with fewer computing resources in the shortest amount of time [64].

## 3. Experiments and Results

The aim of this study is to investigate the effectiveness of MLP classifiers trained with PSO and different potential supervised ML algorithms for developing binary classification models that can be used as diagnostics for heart disease. The Cleveland heart disease dataset is used to evaluate the performance of the algorithms. The data were preprocessed and cleaned, including identifying and handling missing values, categorical variable conversion, and feature scaling using the standardization method.

### 3.1. Experimental Setup

Python [65] was used to conduct all research experiments. The analysis relied on the following libraries and coding packages: (1) Scikit-Learn [66], (2) Pandas [67], (3) NumPy [68], (4) Matplotlib [69], and (5) the PSO optimization library PySwarms [70]. The controlling parameters of the PSO algorithm employed to train the MLP network are summarized in Table 1.

The objective function to be minimized for the MLP network is the the mean squared error (MSE). The MSE is the most basic and widely used objective/loss function. It is calculated as the mean or average squared difference between the true values and the model’s predicted values. The formula for MSE is as follows:

(6) $$\mathrm{MSE}=\frac{1}{n}\sum_{i=1}^{n}{\left(y_{i}-{\hat{y}}_{i}\right)}^{2}$$

where *n* is the number of instances we are testing against, $y_{i}$ is the true value, and ${\hat{y}}_{i}$ is the model’s predicted value.

The swarm size, or the number of particles or candidate solutions, is set to 100. Increasing the swarm size does not always imply that convergence is faster because more particles are searching for the solution. A large swarm necessitates more computation time per epoch, resulting in fewer weight updates for a given training time. A small swarm, on the other hand, will update more frequently; however, with fewer particles, it may escape the solution. Therefore, best practice is to balance by choosing a swarm size that falls somewhere between.

The algorithm terminates when the number of iterations reaches 50 or the objective function value for the particle in the swarm is less than or equal to the predefined error limit. The proposed MLP network structure that was trained using the PSO algorithm had 30 hidden neurons with ReLU as the activation function, and, since heart disease is a binary classification problem, the output layer had one output neuron with sigmoid as the activation function. Ten classifiers were tested to comparatively evaluate the performance of the proposed MLP-PSO model.

In order to find a good model for each of the comparison ML classifiers, a sensitivity study using different hyperparameters of the algorithms was iterated on with grid search. Observing the results of both the training and validation data, the best model is the one with the highest accuracy while avoiding overfitting. All models were tested using five-fold cross-validation and grid search, which iterates on various classifier hyperparameters. The best hyperparameters of each algorithm found by the grid search method are provided in Appendix B.

### 3.2. Performance Evaluation Metrics

**Figure 8:**
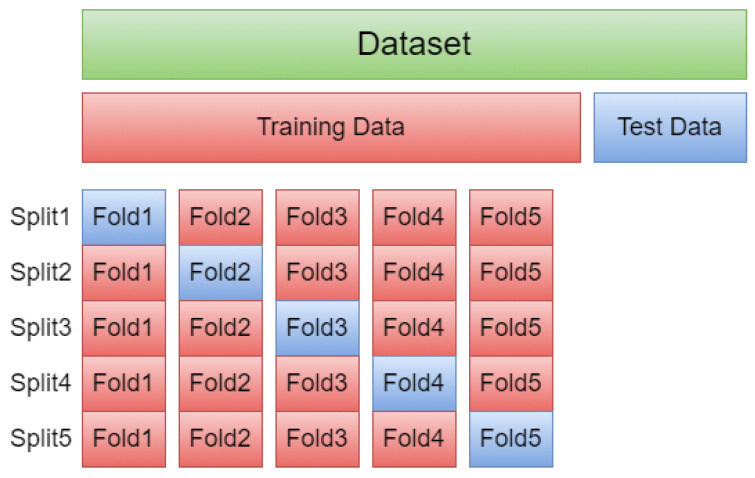
Five-fold cross-validation.

**Figure 1:**
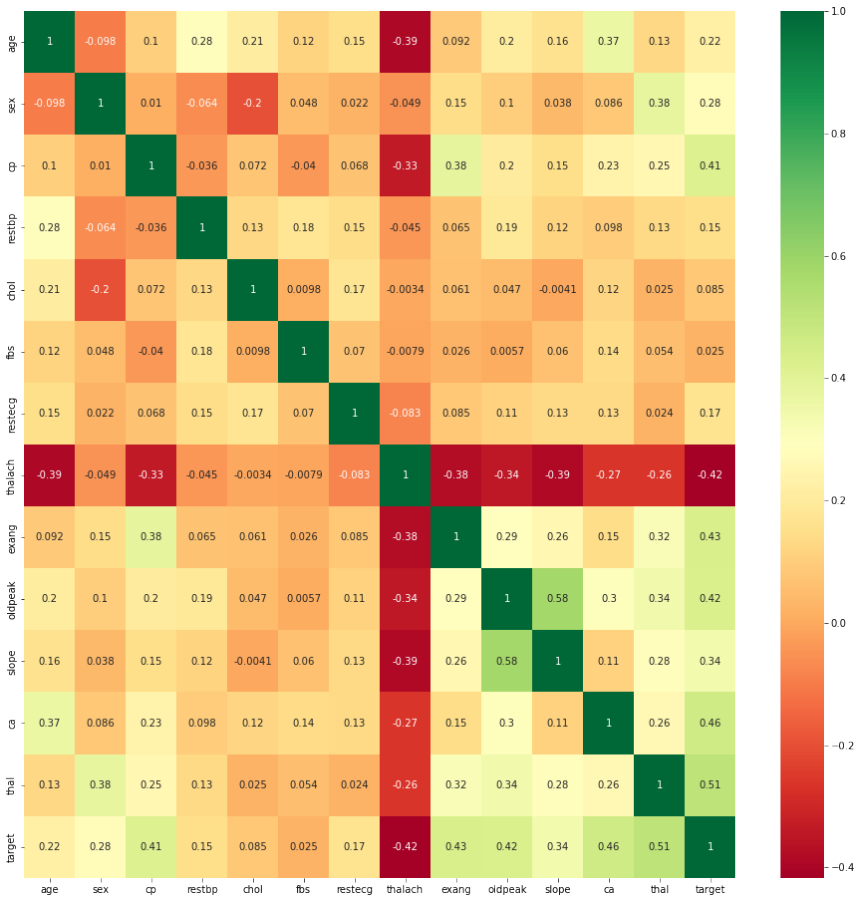
Correlation matrix.

**Figure 2:**
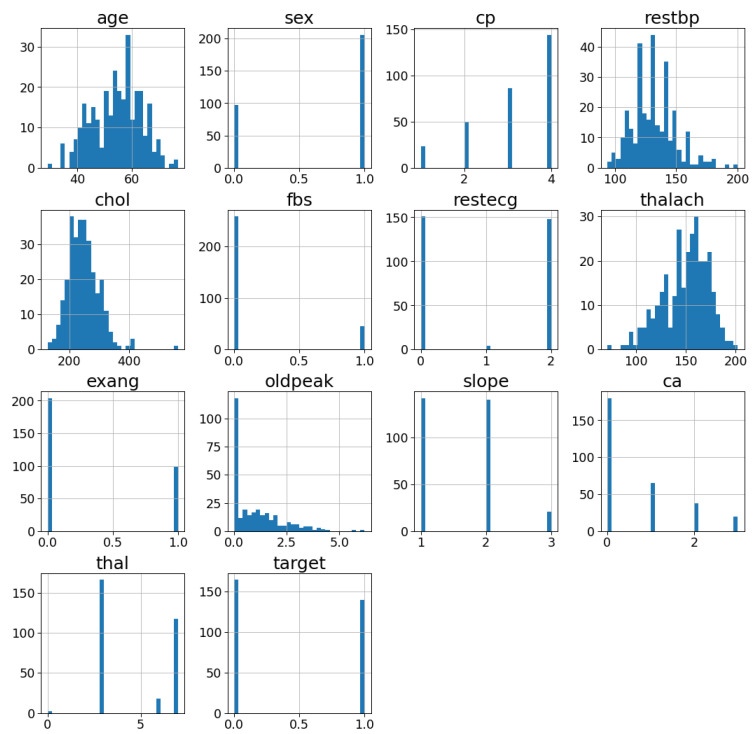
Histogram: A visual representation of the distribution of the Cleveland dataset.

**Figure 3:**
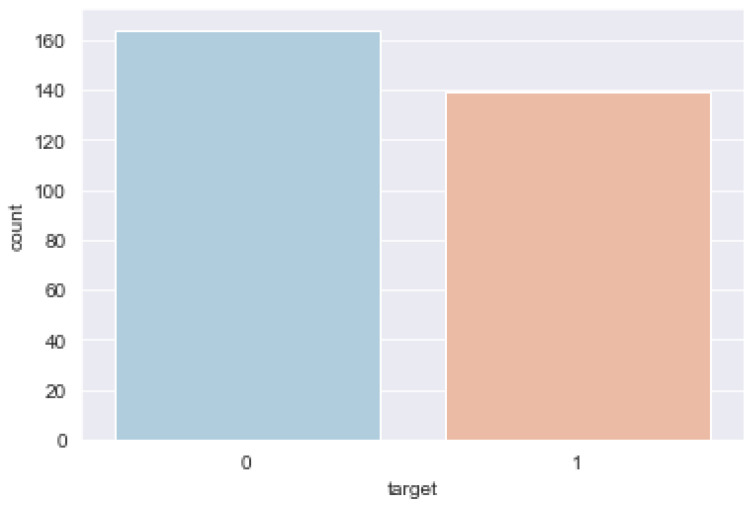
Count of each target class.

**Figure 4:**
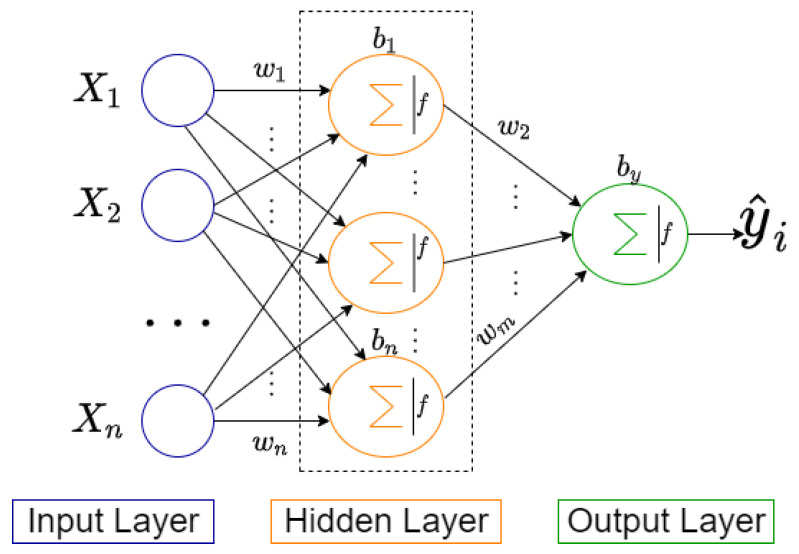
Architecture of a typical three-layer MLP neural network.

**Figure 5:**
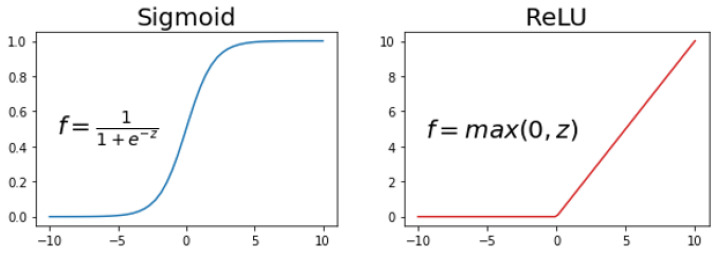
Activation functions: Sigmoid and ReLU.

**Figure 6:**
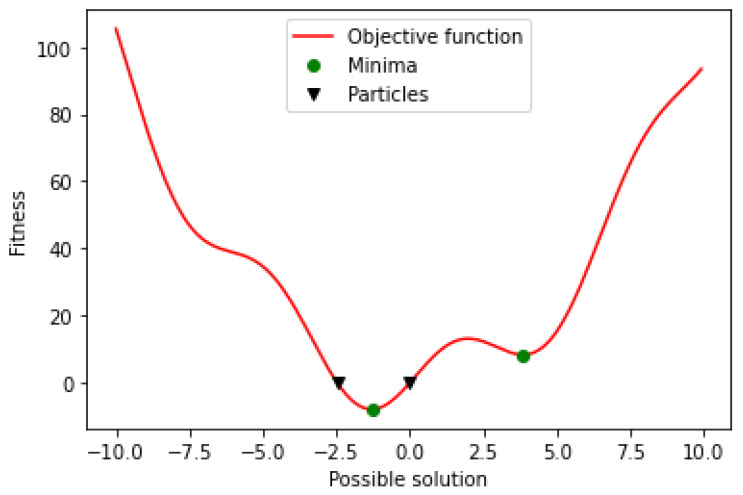
Function minimum.

**Figure 7:**
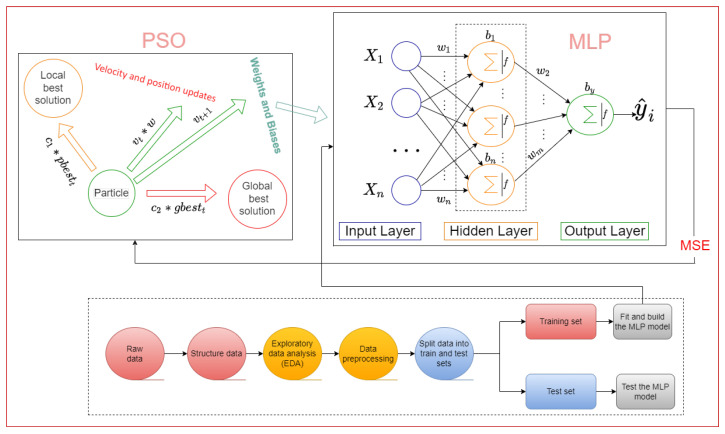
Schematic diagram of the proposed MLP-PSO model for heart disease prediction.

We used 70% of the data for training, while the remaining 30% was used for testing for all models. We used the five-fold cross-validation approach to train the proposed ML models. Five-fold cross-validation is commonly used in applied machine learning to compare and select a model for a given predictive modeling problem because it is simple to implement [71]. The general procedure as depicted in Figure 8 is as follows:

- Randomly shuffle the data.
- Data are split into five groups.
- For each group:
  - The model is trained using four of the folds as training data.
  - The resulting model is tested on the remaining test data to estimate the performance.

We assess a set of performance measures, including the accuracy, precision, recall, and F1 score for each model. These are a set of traditional classification performance measures that are based on the four values of the confusion matrix: True Positives (TP), False Positives (FP), True Negatives (TN), and False Negatives (FN). In addition, the AUC (Area Under The Curve) for the ROC (Receiver Operating Characteristics) curve is graphed. The performance measures are explained as follows:

- TP: the number of positive instances in which the model correctly predicts the presence of heart disease.
- TN: the number of negative instances in which the model correctly predicts the absence of heart disease.
- FP: the number of negative instances in which the model incorrectly predicts the presence of heart disease.
- FN: the number of positive instances in which the model incorrectly predicts the absence of heart disease.
- Accuracy: the ratio of correctly predicted instances to all instances, which is defined as follows:
  (7) $$\mathrm{Accuracy}=\frac{\mathrm{TP}+\mathrm{TN}}{\mathrm{TP}+\mathrm{TN}+\mathrm{FP}+\mathrm{FN}}$$
- Precision: the ratio of correctly predicted positive instances to the total number of predicted positive instances, which is defined as follows:
  (8) $$\mathrm{Precision}=\frac{\mathrm{TP}}{\mathrm{TP}+\mathrm{FP}}$$
- Recall: the ratio of correctly predicted positive instances to all instances in the actual class (presence), which is defined as follows:
  (9) $$\mathrm{Recall}=\frac{\mathrm{TP}}{\mathrm{TP}+\mathrm{FN}}$$
- F1 score: the weighted average of Precision and Recall, which is defined as follows:
  (10) $$F1\;\mathrm{score}=2\times \frac{\mathrm{Precision}\times \mathrm{Recall}}{\mathrm{Precision}+\mathrm{Recall}}$$
- AUC-ROC Curve: ROC is a probability curve, and AUC represents the measure of a model to distinguish between classes. It tells how much the model is capable of distinguishing between classes. The higher the AUC, the better the model is at distinguishing between the classes. The ROC curve is plotted with the TPR on the *y*-axis against the FPR on the *x*-axis. The TPR is a synonym for recall, and FPR is defined as as follows:
  (11) $$\mathrm{FPR}=\frac{\mathrm{FP}}{\mathrm{FP}+\mathrm{TN}}$$

### 3.3. Results and Discussion

This study aimed to develop a diagnostic for heart disease using machine-learning technology. Many different models, including our proposed MLP-PSO, were trained, optimized, and evaluated. Table 2 shows the experimental results for all the predictive models on the Cleveland heart disease dataset. The accuracy, AUC, precision, recall, and F1 score were used to evaluate each model using five-fold cross-validation.

The results show that the testing performance of our proposed MLP-PSO model outperformed all other models in terms of all the performance measures evaluated. This indicates that PSO is robust in converging to an optimal set of weights and biases for the MLP model. As a result, the MLP model was able to find a desirable solution to the problem.

By observing the experimental results, we can also see that the GaussianNB and SVM classifiers are performing well; however, when comparing the two models, GaussianNB performed better. It is also clear from the results that the Gradient Boosting classifier had the worst testing performance among all the models. We can also see that MLP with BP performed well compared to GaussianNB and SVM but not as well as MLP-PSO. This is because BP is prone to becoming stuck in local minima and is sensitive to the values used for the initial weights, the learning rate, and, if used, the momentum.

These are critical factors to consider when developing an MLP model. In some cases, a small change in any of these values has a significant impact on the predictive accuracy of the MLP model being trained. On the other hand, PSO is much less sensitive to its free parameters and can jump out of local minima or the weights can be reinitialized to start looking in a new area of the search space; therefore, it does well at minimizing the error if run for long enough. The drawback, however, is that PSO took longer to converge (226.0 s) to find the solution versus BP (3.0 s). For further analysis, the ROC curves for all classifiers are provided in Appendix C. MLP-PSO classifier’s ROC curve was the most stable and accurate, whereas the ROC curve of the Gradient Boosting classifier was the most unsteady.

## 4. Conclusions and Future Directions

Cardiovascular disease can be difficult to detect even for the most experienced healthcare providers. AI tools built with ML algorithms can be used to correctly diagnose disease and predict patients’ future health. Neural networks remain the most promising and widely used ML technique, particularly for tasks including disease detection and treatment recommendations. Neural networks have the ability to train themselves continually and to significantly improve their performance. However, neural networks have certain limitations. One of the most common is associated with the training method. The BP algorithm that is often used cannot guarantee an optimal solution. PSO algorithms are an effective optimization technique that can optimize the training process of neural networks.

In this paper, we proposed an MLP neural network trained with PSO for heart disease detection. We also investigated various potential ML algorithms for developing intelligent diagnostic heart disease systems. Study experiments were analyzed using the Cleveland dataset. We focused mainly on attempting to distinguish the presence versus the absence of heart disease using 13 features. The experiments demonstrated that the proposed MLP-PSO outperformed all other algorithms, achieving an accuracy of 84.61%. The findings demonstrated that the MLP-PSO model can assist healthcare providers in more accurately diagnosing patients and recommending better treatments. Overall, the use of neural networks trained with PSO appears promising in the detection of heart disease.

In the future, the proposed MLP-PSO will be evaluated and analyzed further on a variety of medical-based datasets to demonstrate its capability in medical diagnosis and healthcare. Furthermore, optimal typologies for the MLP and other deep neural network models will be tuned and optimized using PSO. The careful design of neural network typologies is the key to their success in solving problems. The best neural network topology for a given problem is frequently determined manually, which can be time-consuming and requires an expert with in-depth domain knowledge. The PSO optimization technique can provide a powerful tool for topology design automatically. Other suggestions for future research include collecting findings and comparing the performance of various swarm algorithms using the Cleveland Heart Disease dataset and other available datasets.

## Figures and Tables

**Table 1:** PSO algorithm controlling parameters.

Parameter	Value
Swarm size	100
Iterations	50
$r_{1}$	0.4
$r_{2}$	0.5
$c_{1}$	0.5
$c_{2}$	0.3
*w*	0.9

**Table 2:** Test performance comparison using various performance evaluation metrics.

Algorithm	Accuracy	AUC	Precision	Recall	F1 Score
MLP-PSO Classifier	0.846	0.848	0.808	0.883	0.844
Decision Tree Classifier	0.758	0.756	0.775	0.704	0.738
Extra Trees Classifier	0.769	0.766	0.810	0.681	0.740
GaussianNB Classifier	0.824	0.821	0.868	0.750	0.804
Gradient Boosting Classifier	0.714	0.712	0.725	0.659	0.690
KNN Classifier	0.780	0.777	0.815	0.704	0.756
Logistic Regression Classifier	0.813	0.808	0.909	0.681	0.779
MLP Classifier with BP	0.802	0.799	0.861	0.704	0.775
Random Forest Classifier	0.791	0.787	0.857	0.681	0.759
SVM Classifier	0.813	0.809	0.885	0.704	0.784
XGB Classifier	0.769	0.766	0.810	0.681	0.740

**Figure A1:**
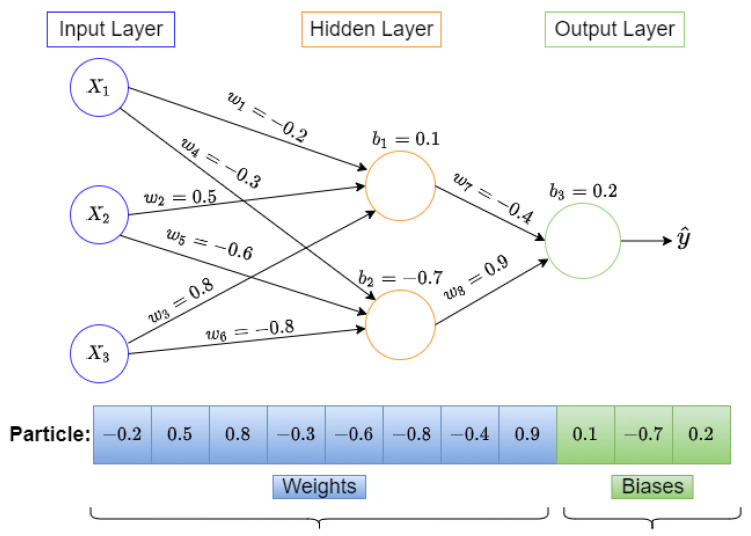
Encoding the MLP weights and biases in a particle.

**Figure A2:**
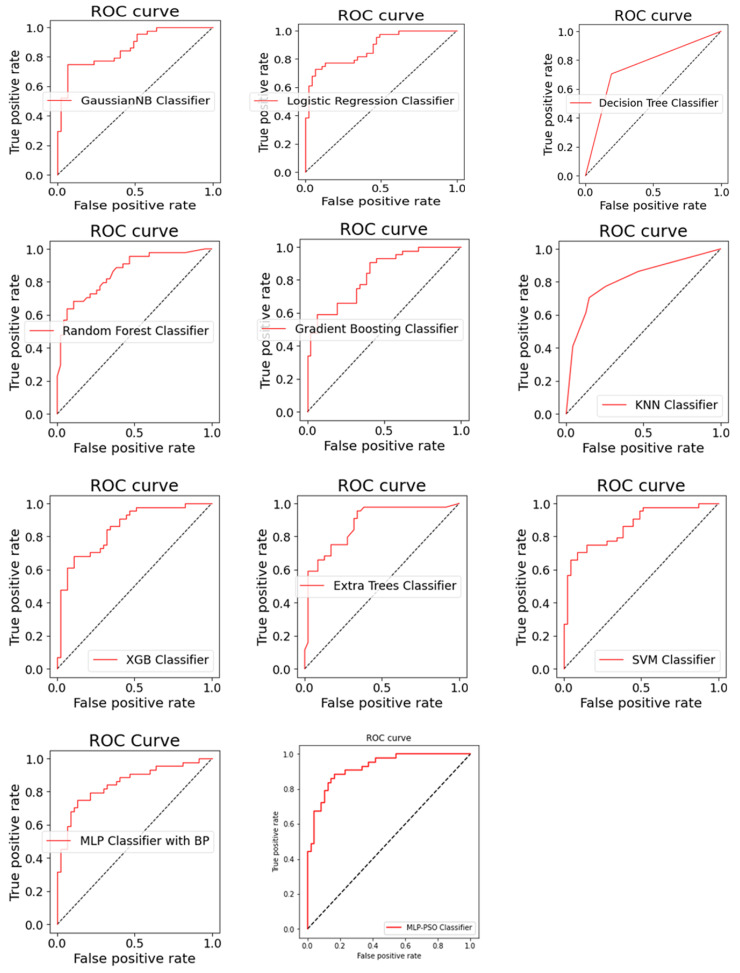
ROC curves.

## Data Availability

https://archive.ics.uci.edu/ml/datasets/heart+disease.

## Appendix A. MLP Training Problem Representation Using PSO

To begin using PSO, the problem domain must first be represented as a particle. Each particle in the swarm represents a potential solution. We assume that we want to find the best set of weights and biases for the MLP network depicted in Figure A1. The initial weights and biases in the MLP are initialized to small random values in the range [−1, 1]. The set of weights and biases can then be represented by a vector (particle), with a real number corresponding to each weight and bias. In total, there are 11 weight and bias parameters in the MLP shown in Figure A1, and thus the vector length is 11. If the swarm has a population of 100, then each generation (or iteration) contains 100 vectors, each of which is a potential MLP Network.

## Appendix B. Model Hyperparameters

**Table A1:** Model hyperparameters and their optimal values.

Algorithm	Parameters	Algorithm	Parameters
**Decision Tree Classifier**	criterion = ‘Gini’ min_samples_leaf = 1 min_samples_split = 2 max_features = log2(n_features) splitter = ‘best’	**Logistic Regression Classifier**	C = 1.5 fit_intercpet = ‘True’ Penalty = ‘12’
**Extra Trees Classifier**	Criterion = ‘Gini’ min_samples_leaf = 1 min_samples_split = 2 max_features = log2(n_features) n_estimators = 100	**MLP Classifier with BP**	hidden_layer_size = 30 activation = ‘relu’ learning_rate = ‘adaptive’ momentum = 0.9 optimizer = ‘adam’
**GaussianNB Classifier**	var_smoothing = 1 priors = ‘None’	**Random Forest Classifier**	max_features = sqrt (n_features) n_estimators = 1000 criterion = ‘Gini’
**Gradient Boosting Classifier**	criterion = ‘friedman_mse’ loss = ‘deviance’ learning_rate = 0.1 n_estimators = 100 max_depth = 3 max_features = log2(n_features)	**SVM Classifier**	C = 1 coef0 = 10 degree = 3 gamma = 0.1 kernel = ‘rbf’
**KNN Classifier**	metric = ‘minkowski’ n_neighbors = 5 p = 2 weights = ‘uniform’	**XGB Classifier**	colsample_bytree = 0.6 reg_lambda = 0.68 reg_alpha = 72 max_depth = 15 min_child_weight = 1 gamma = 3.27 n_estimators = 300

## Appendix D. Source Code

All source code for this study is available under the Apache 2.0 license and can be obtained by contacting the corresponding author at aalbatai@norwich.edu.
